# Faster inference of complex demographic models from large allele frequency spectra

**DOI:** 10.1093/bioinformatics/btag511

**Published:** 2026-07-11

**Authors:** Enes Dilber, Jiatong Liang, Junyan Tan, Jonathan Terhorst

**Affiliations:** Department of Statistics, University of Michigan, Ann Arbor, MI 48109, United States; Department of Statistics, University of Michigan, Ann Arbor, MI 48109, United States; Department of Statistics, University of Michigan, Ann Arbor, MI 48109, United States; Department of Statistics, University of Michigan, Ann Arbor, MI 48109, United States

## Abstract

**Motivation:**

Demographic inference from the joint site frequency spectrum is limited by computation when many populations or many samples are analyzed.

**Results:**

We present momi3, a JAX-based method for inferring complex demographic models from large allele frequency spectra. It supports continuous migration, GPU execution, automatic differentiation, standardized demographic model input, and genealogical pruning. These changes yield speedups up to 1000× over existing methods and enable analysis of archaic admixture models using hundreds of human genomes.

**Availability and implementation:**

momi3 is implemented in Python/JAX as part of demestats. Source code is available at https://github.com/jthlab/demestats; documentation is available at https://demestats.readthedocs.org.

## 1 Introduction

It is now widely appreciated that patterns of population genetic variation can be used to recover detailed information about the timing and magnitude of changes in effective population size, migration, and admixture events, a pursuit known as *demographic inference* (Marchi *et al.* 2021). Recently, the amount of data available for performing demographic inference has experienced substantial growth, along three different dimensions. First, the number of samples collected from individual populations continues to grow at a fast rate, due to the ever-decreasing cost of sequencing genomes. Second, the number of populations that have been sampled has itself also sharply increased (The 1000 Genomes Project Consortium 2015, Mallick *et al.* 2016), owing in part to growing recognition of the scientific and ethical importance of sampling underrepresented groups. Finally, as a result of impressive technical achievements in ancient DNA extraction and analysis (Green *et al.* 2010, Meyer *et al.* 2012, Prüfer *et al.* 2014), we now have access to the genomes of individuals who lived at many different points in time as well.

New data sources should, in principle, enable us to answer increasingly precise and subtle questions about evolutionary history. However, in order to realize such a goal, lingering technical obstacles must be surmounted. Evaluating the likelihood of raw sequence data under completely realistic biological and evolutionary models is infeasible, even for small sample sizes (Li and Stephens 2003, Sheehan *et al.* 2013, Rasmussen *et al.* 2014, Terhorst *et al.* 2017). Therefore, approximations or data reduction strategies have to be employed in order to scale demographic inference methods up to the size of modern data sets.

In this article, we focus on one such strategy, which is to first summarize genetic variation data into a low-dimensional tensor, and then find an evolutionary model which would have generated a similar statistic. The summary statistic we focus on is known as the (joint) site frequency spectrum (JSFS). With *n* exchangeable samples from a panmictic population, the site frequency spectrum (SFS) is an (n+1)-dimensional vector that counts the number of singletons, doubletons, tripletons, etc. that were observed, and the first and last entries are monomorphic sites that are typically not considered. The joint SFS is its multidimensional generalization: if there are $n_{1},\ldots ,n_{p}$ samples from each of populations (or “demes”), the JSFS is a tensor in $Z_{\geq 0}^{\left(n_{1}+1)\times \cdots \times (n_{p}+1\right)}$.

Since the size of the JSFS grows exponentially in the number of demes, state-of-the-art SFS-based demographic inference methods like ∂a∂i (Gutenkunst *et al.* 2009), momi2 (Kamm *et al.* 2020), or moments (Jouganous *et al.* 2017) are limited to analyzing, in a practical amount of time, perhaps several dozen individuals sampled from about five demes. Unfortunately, this falls well short of the quantity of data that is now available. Wohns *et al.* (2022), e.g. recently published a unified dataset containing over 3600 human genomes from 215 populations, far exceeding what any of these methods can analyze. As a result, researchers face the unpleasant choice of either throwing away information to get an answer quickly, or waiting a large amount of time to perform a complete analysis.

To address this shortcoming, we have developed a new, scalable method called momi3 for inferring complicated demographic models using modern data sets. It features many improvements over its predecessor momi2. First, the internal code has been completely rewritten using JAX, a neural network library designed for performing large-scale machine learning on graphs (Bradbury *et al.* 2018). This brings several benefits, including end-to-end automatic differentiability, just-in-time (JIT) compilation, and the native ability to run on an accelerator (GPU/TPU). Second, support for modeling and inferring continuous migration has been added, thus lifting one of the main technical restrictions of momi2. Third, momi3 features a novel pruning technique which can drastically reduce computational requirements for analyzing complex, multi-population demographic models. Fourth, we have eliminated large amounts of custom API in favor of standardized, community-developed libraries: tskit for storing and accessing genotypes; msprime (Baumdicker *et al.* 2022) for conducting simulations; and demes (Gower *et al.* 2022) for demographic model specification and visualization. Taken together, these advances enable momi3 to fit models containing hundreds of sampled individuals spread across many populations, in a computationally efficient and intuitive manner.

## 2 Background

Formally, we want to solve the following estimation problem: given allele count data $\Xi \in Z_{\geq 0}^{\left(n_{1}+1)\times \cdots \times (n_{p}+1\right)}$ from *p* different populations, find a demographic model Θ existing in some model class M that maximizes the likelihood of the data:


$$\hat{\Theta }=\underset{\Theta \in M}{\text{argmax}}\;\ell (\Theta |\Xi )$$

Under further assumptions (namely, linkage equilibrium and infinite sites) needed to render the problem tractable, it can be shown (Bhaskar *et al.* 2015) that the maximum likelihood problem is equivalent to minimizing the Kullback–Leibler divergence between the observed frequency spectrum, and its expected value $E_{\Theta }\Xi$ (when both are normalized to form discrete probability distributions):


(1) $$\hat{\Theta }=\underset{\Theta \in M}{\text{arg min }}d_{\text{KL}}(\Xi \;∥\;E_{\Theta }\Xi ).$$

### 2.1 Prior work

Given Ξ and $E_{\Theta }\Xi$, computing the KL divergence is trivial; the difficulty of the problem lies entirely in evaluating the expectation. At least three different strategies have been proposed. First, simulation-based methods (Excoffier and Foll 2011, Excoffier *et al.* 2013, 2021) use Monte Carlo estimation to numerically approximate $E_{\Theta }\Xi$. These have the advantage of being very flexible and requiring fewer simplifying assumptions; hence, the class of models [the M in Equation (1)] is larger, and includes any process that can be forward-simulated. However, as Monte Carlo methods, they suffer from the curse of dimensionality, and encounter trouble when the number of populations *p* is large. In particular, in large samples the JSFS tensor Ξ is extremely sparse (see examples below), so, unless many iterations are used, the Monte Carlo estimate of $E_{\Theta }\Xi$ has a different sparsity pattern, and the objective function (1) is not even defined.

As an alternative to simulation-based approaches, several exact methods have also been proposed. There are basically two approaches. Diffusion-based methods work by solving a system of differential equations which characterize the distribution of a biallelic variant over time in the infinite population limit. Two well-known implementations of this technique are the programs ∂a∂i (Gutenkunst *et al.* 2009, Gutenkunst 2021) and moments (Jouganous *et al.* 2017). The second class of methods uses coalescent theory to compute $E_{\Theta }\Xi$. This line of work originates in a seminal paper by Griffiths and Tavaré (1998), who showed how to compute the expected frequency spectrum for a single population whose historical size varied over time. Chen (2012, 2013) later extended this result to multiple, non-admixing populations, as did Kamm *et al.* (2017), using different ideas which led to a much faster algorithm. Most recently, Kamm *et al.* (2020) further generalized the method to the case of admixed populations in the program momi2.

The coalescent and diffusion approaches possess different, and somewhat complementary, advantages and disadvantages. The runtime of the latter is independent of sample size, and the diffusion can more easily incorporate non-neutrality and migration. However, convergence issues can arise when integrating the differential equations, and they are limited in the number of populations they can analyze—the original version of ∂a∂i could handle up to three; this was later increased to five using GPUs (Gutenkunst 2021). moments removes some of these limitations by solving an alternative system of differential equations, but is also limited to no more than five populations, and its runtime scales with sample size. Another important difference between the approaches emerges in large data sets: diffusion methods essentially solve for the entire joint frequency spectrum, even though most entries are not observed in real data, resulting in much wasted computation. For their part, coalescent-based methods are able to compute individual SFS entries, but have a computational requirement that grows with the number of samples, and cannot easily accommodate selection or continuous migration.

### 2.2 New contributions

momi3 is a significantly updated version of our earlier method momi2 which is oriented towards analyzing modern, large-scale population genetic datasets in a timely fashion. In addition to being much faster than existing methods, momi3 is able to combine the advantages of both the diffusion and coalescent approaches to demographic inference using the JSFS. In this section, we briefly summarize these technical advances. A fully rigorous explanation requires much additional notation, and is not necessary to understand the rest of the paper, so we defer it to the Supplementary Material, available as supplementary data at *Bioinformatics* online.

#### 2.2.1 Continuous migration

momi2 allowed for specifying (subject to memory and computation constraints) arbitrary numbers of “pulse” admixture events between populations. In this type of event, each surviving ancestral lineage present in a particular source population migrates independently with probability *p* to a specified destination population. However, mathematical difficulty prevented us from incorporating the continuous flow of migrants between populations. In momi3, we have added support for continuous migration, thus lifting one of the main technical restrictions of momi2. To achieve this, we build on recent work by Jouganous *et al.* (2017) who used a moment-based approach to speed up the diffusion calculations necessary for computing the expected SFS forwards in time. The end result is that momi3 is able to analyze demographies containing any combination of pulse admixtures and continuous migration.

#### 2.2.2 Genealogical pruning

A second improvement over momi2 concerns the computation of likelihoods when there are many samples. For a given variant, momi2 does this by integrating over all possible genealogies on which a variant could have arisen. However, in large samples, this integral often includes portions of the genealogical state space that are extremely unlikely, and contribute negligibly to the end result. For example, in a demography that features many pulse admixtures, momi2 expends computation to consider the scenario where *every* lineage migrates from destination to source population at *every* admixture event. Similarly, in a population which has experienced recent exponential growth, i.e. most human populations, momi2 allows for the possibility that *none* of the present-day samples have reached common ancestors, even thousands of generations back into the past.

To improve the efficiency of momi3, we implemented a pruning strategy which removes unlikely genealogies from the integration, thereby lessening both the memory and computational requirements of analyzing large samples. In our current implementation, we focused on two types of events that are likely to result in such a reduction. The first is transitioning (“lifting”) a partial likelihood backwards for large amounts of coalescent time. In large samples, the number of remaining ancestral lineages decreases precipitously, so by reasoning about the coalescent ancestral process, we can confine attention only to genealogies which feature a likely reduction in the number of ancestral lineages (Tavaré 1984).

A similar approach can be used to lessen the computational requirements for models involving admixture. Going backwards in time, a pulse admixture event causes a population *v* (say) to split into two parent populations of sizes $n_{v}$ each, because it is possible that as few as none, or as many as all, of the lineages descended from either parent population. Let *p* be the expected fraction of lineages in *v* that came from parent 1, with the remainder coming from parent 2. The total number of lineages *k* in *v* that came from parent 1 is distributed $k∼\mathrm{Binomial}(n_{v},p)$. Hence, by considering the tail probabilities of the binomial cumulative distribution function (which are exponentially small in $\left|k-n_{v}p\right|$), we can again restrict attention to a typically small range of possible *k* that have non-negligible mass.

The overall approximation depends on a tuning parameter, 0<ϵ<1, which controls the amount of ignored probability mass in the computation—if ϵ=0.05 (say), then we restrict the integration to a set of genealogies that comprise 95% of the mass. Increasing ϵ trades accuracy for computation time. We explore this in more detail in simulations below. Finally, we note that, as currently implemented, the procedure requires that at least some amount of time elapse before populations interact with each other in order to achieve computational savings. In particular, it cannot currently be used to simplify computations for large populations that continuously exchange migrants all the way up to the present.

#### 2.2.3 Implementation improvements

momi3 has been rewritten from the ground up using modern machine learning libraries designed to perform tensor computations on graphs. We selected the differentiable programming language JAX (Bradbury *et al.* 2018) due to its combination of speed, ease-of-use, and support for the Numpy API already utilized by momi2. This brings several benefits. First, momi3 natively supports automatic differentiation and JIT compilation, leading to speedups as high as 1000-fold, as shown below. Second, the implementation seamlessly runs on CPU, GPU, as well as Google tensor processing (TPU) hardware, requiring no more than a command-line switch to select between the different backends. Third, the speed and stability of momi3 will continue to improve as JAX matures. For example, while we were developing the method, support for sparse tensor computations in JAX improved considerably, leading to performance gains in momi3 with no additional effort on our part.

Readers should keep in mind that this approach requires a one-time compilation step, which can require several minutes for complex demographic models. This step must be repeated any time the structure of the demography changes. Typically, the cost of compilation will be amortized over many evaluations of the likelihood function and/or its gradient, however this may not always be true. One scenario where compilation time might become excessive is when searching over many topologically distinct demographic models, since recompilation would need to happen for each new topology. We discuss the cost of compilation in the simulation examples below.

Except in simple cases, exact analytical gradients are not available for this problem. Although finite-difference schemes can yield numerical approximations, the use of automatic differentiation in JAX mitigates numerical instabilities and approximation errors associated with such methods. The optimization procedure is highly sensitive to the underlying demographic model and the specific parameters being inferred. In our simulation studies, we observe that, under complex demographic scenarios, the likelihood surface is strongly non-convex, necessitating the use of optimization algorithms with multiple random initializations. Our results indicate that increasing the number of random starting points systematically improves the quality and robustness of the inferred parameter estimates.

#### 2.2.4 User interface improvements

Finally, we have made momi3 easier to use by relying on community-maintained libraries and standards wherever possible. We have eliminated momi2’s custom API for demographic model specification in favor of demes (Gower *et al.* 2022). Users can perform inference simply by providing demography model in demes format, along with the data, and model estimates can also be returned as demes-formatted demographies. Similarly, momi3 can directly interface with msprime (Baumdicker *et al.* 2022) for demographic model simulation and bootstrapping, and can directly read succinct tree sequence data (Kelleher *et al.* 2016) using tskit.

## 3 Results

### 3.1 Performance of momi3 vs. existing methods

**Figure 1:**
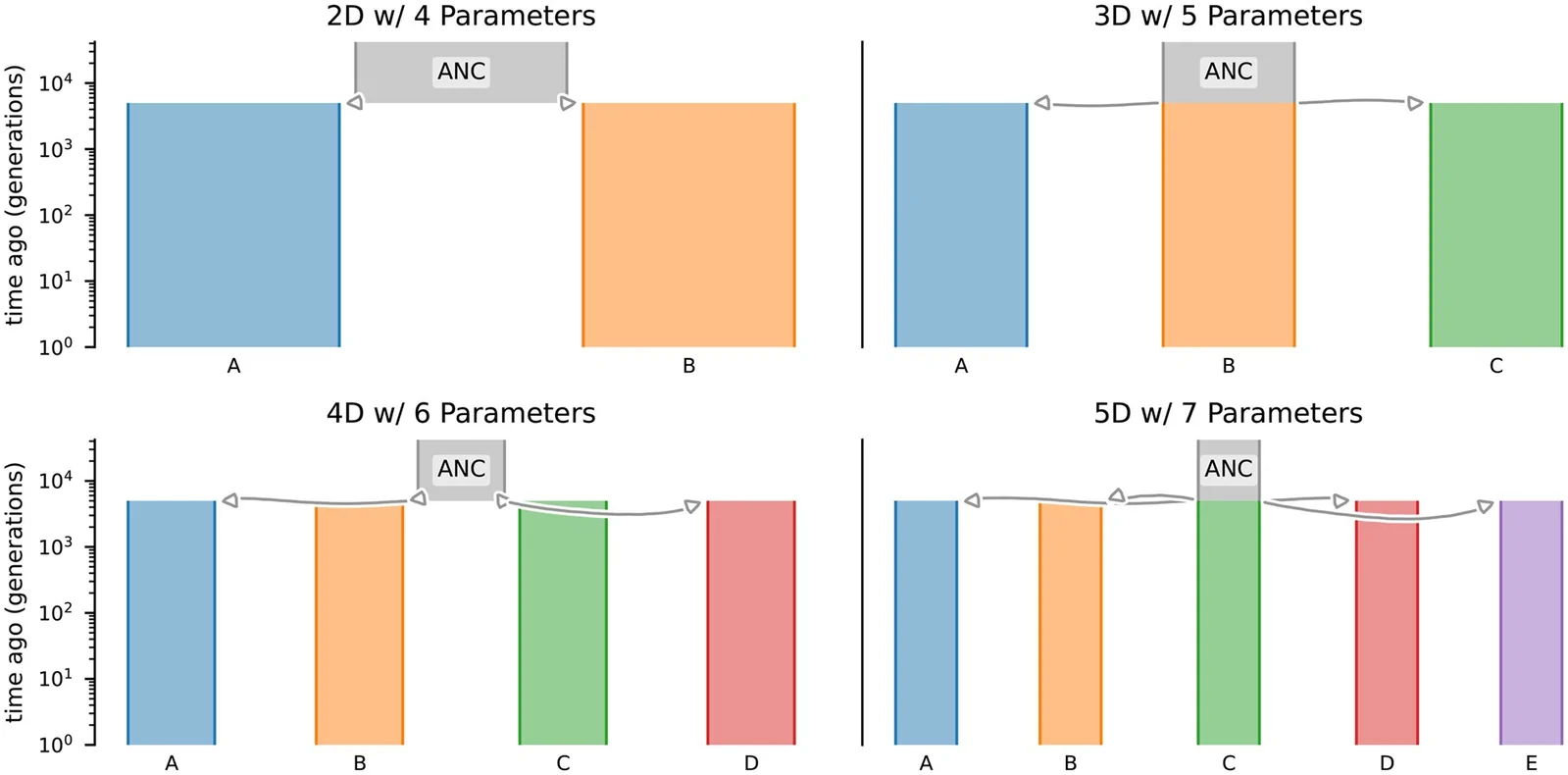
Constant population size demographies. All demes have effective population size $2N=5\times 10^{3}$ and diverged $5\times 10^{3}$ generations ago. Parameters are shown in Table 1.

**Figure 2:**
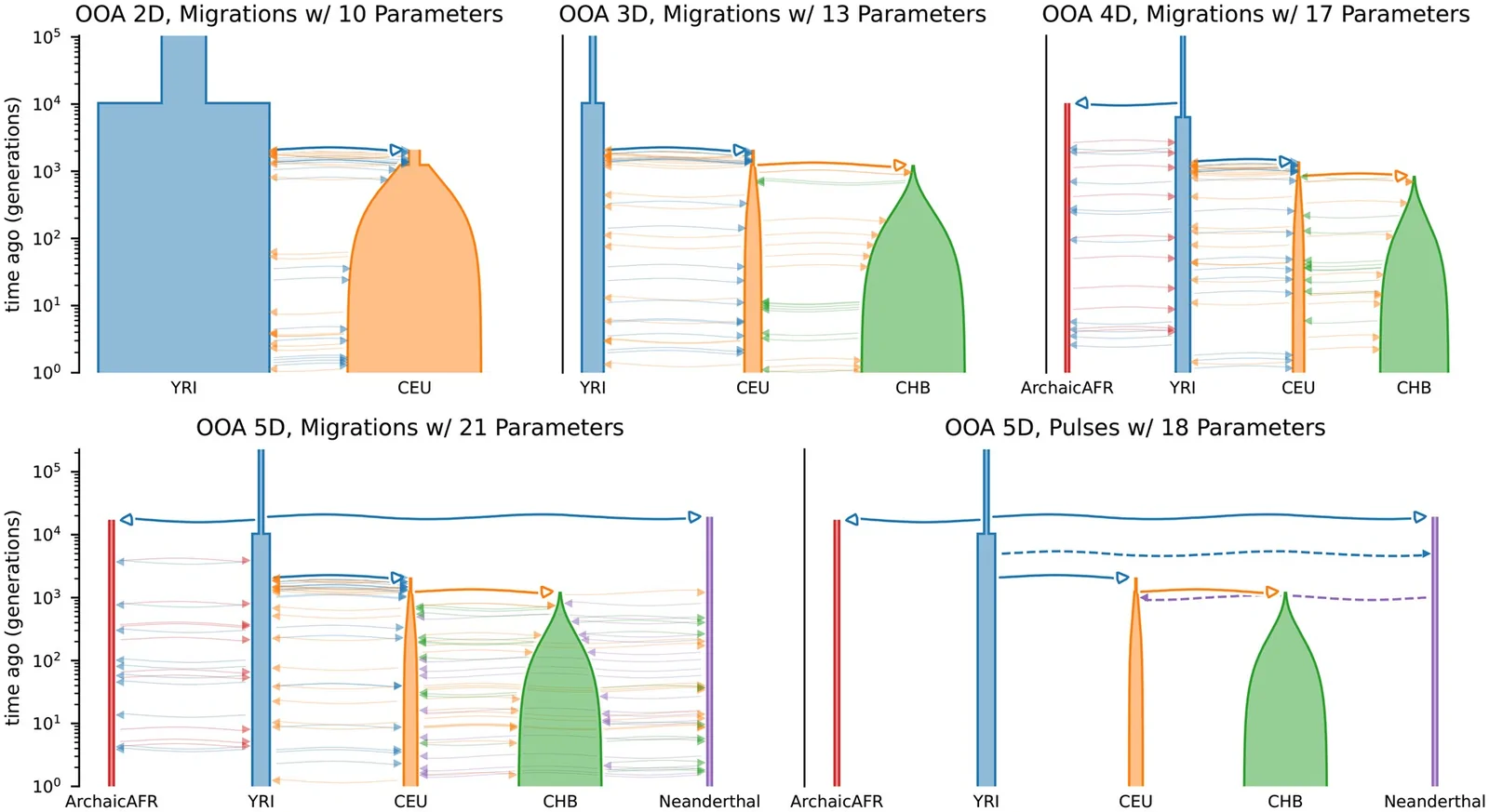
Out-of-Africa demographies. Parameters are shown in Table 2.

We compared momi3 to our earlier software momi2, as well as to moments, which is the state-of-the-art diffusion-based method for this problem. We measured both runtime and accuracy. Benchmarks were carried out using nine different demographies. The models denoted *xD*, for x∈{2,3,4,5}, are demographies containing *x* isolated populations with no migration or admixture, and constant population size. (Population size refers to coalescent effective population size throughout.) The OOA models are Out-of-Africa models estimated previously by Ragsdale and Gravel (2019). Only for the model with pulse migration, we added two pulse migrations to the demography tree for testing purposes. Visualizations of each of the models are shown in Figs 1 and 2. Summaries of all of the parameters for each model are in Tables 1 and 2. All of the simulations used a simulated chromosome of length 100Mbp.

**Table 1:** Parameters in constant population size models.

Parameters	2D	3D	4D	5D
Size of ANC	✓	✓	✓	✓
Size of A	✓	✓	✓	✓
Size of B	✓	✓	✓	✓
Size of C		✓	✓	✓
Size of D			✓	✓
Size of E				✓
Split time	✓	✓	✓	✓
Total number of parameters	4	5	6	7

**Table 2:** Parameters in out-of-Africa models.

Parameters	OOA 2D	OOA 3D	OOA 4D	OOA 5D	OOA 5D
	Migrations	Migrations	Migrations	Migrations	Pulses
Ancient size of YRI	✓	✓	✓	✓	✓
Recent size of YRI	✓	✓	✓	✓	✓
Size of Neanderthal				✓	✓
Size of ArchaicAFR			✓	✓	✓
Ancient size of CEU	✓	✓	✓	✓	✓
Size of CEU just before exp growth	✓	✓	✓	✓	✓
Recent size of CEU	✓	✓	✓	✓	✓
Size of CHB just before exp growth		✓	✓	✓	✓
Recent size of CHB		✓	✓	✓	✓
Migration rate between YRI and ArchaicAFR			✓	✓	
Migration rate (1st) between YRI and CEU	✓	✓	✓	✓	
Migration rate (2nd) between YRI and CEU	✓	✓	✓	✓	
Migration rate between CEU and Neanderthal				✓	
Migration rate between CHB and CEU		✓	✓	✓	
Migration rate between CHB and Neanderthal				✓	
CHB–CEU split time & recent migration start time	✓	✓	✓	✓	✓
Time of pulse migration from Neanderthal to CEU					✓
CEU–YRI split time	✓	✓	✓	✓	✓
Time of pulse migration from YRI to Neanderthal					✓
Start time of YRI–ArchaicAFR migration			✓	✓	
Size change time of YRI	✓	✓	✓	✓	✓
ArchaicAFR–YRI split time			✓	✓	✓
Neanderthal–YRI split time				✓	✓
Pulse migration from Neanderthal to CEU					✓
Pulse migration from YRI to Neanderthal					✓
Total number of parameters	10	13	17	21	18

#### 3.1.1 Runtime and memory consumption

First we studied the time needed for each method to evaluate the log-likelihood of a particular model, and/or its gradient. For the CPU-based benchmarks, we ran all methods on unloaded cluster nodes containing Intel Xeon Gold 6254 CPUs. Since all the CPU-based methods can use multiple cores, we allocated 15 cores to each method when performing the evaluation (with one additional core occupied by the benchmarking code itself.) For the GPU runs involving momi3, we used a single NVIDIA Tesla V100 GPU.

Table 3 contains the runtimes for each method under these settings. The runtime of likelihood calculation can be seen at the column “Runtime ℓ” and likelihood+gradient calculation in “Runtime ∇ℓ.” We calculated the gradients for each parameter in the given model. We used the built-in automatic differentiation capabilities of momi2 and momi3, while for moments, we relied on numerical differentiation as implemented in their software package.

**Table 3:** Computation time of the likelihood (ℓ) and the gradient (∇ℓ), along with total variation (TV) from the reference method, for several demographic models.^a^

Demographic model	*n*	Method	**Runtime** ℓ	**Runtime** ∇ℓ	TV
2D w/4 Parameters	**200**	Moments	0.94	5.61	3.7e−06
		momi2	0.71	1.94	–
		momi3 (CPU)	0.41	0.65	2.3e−07
		momi3 (GPU)	**0.06**	**0.08**	**2.1e−07**
3D w/5 Parameters	**200**	Moments	77.75	544.68	5.4e−06
		momi2	3.99	12.33	–
		momi3 (CPU)	2.10	3.81	2e−07
		momi3 (GPU)	**0.40**	**0.50**	**1.8e−07**
4D w/6 Parameters	**5**	Moments	0.23	1.81	8.2e−06
		momi2	0.04	0.14	–
		momi3 (CPU)	**0.01**	**0.01**	8.3e−08
		momi3 (GPU)	0.01	0.01	**7.6e−08**
5D w/7 Parameters	**5**	Moments	1.51	13.71	9.1e−06
		momi2	0.10	0.32	–
		momi3 (CPU)	**0.02**	**0.02**	8.8e−08
		momi3 (GPU)	0.02	0.02	**7.4e−08**
OOA 2D, Migrations w/10 Parameters	**20**	Moments	0.25	7.71	–
		momi3 (GPU)	**0.16**	**0.81**	**0.0049**
OOA 3D, Migrations w/13 Parameters	**20**	Moments	**1.59**	94.33	–
		momi3 (GPU)	19.45	**93.76**	**0.027**
OOA 4D, Migrations w/17 Parameters	**5**	Moments	2.23	187.75	–
		momi3 (GPU)	**0.51**	**1.62**	**0.013**
OOA 5D, Migrations w/21 Parameters	**5**	Moments	20.42	2699.14	–
		momi3 (GPU)	**8.98**	**28.34**	**0.034**
OOA 5D, Pulses w/18 Parameters	**5**	Moments	5.73	113.96	6.4e−06
		momi2	0.07	0.30	–
		momi3 (CPU)	0.02	0.06	**1.3e−07**
		momi3 (GPU)	**0.02**	**0.02**	1.6e−07
OOA 5D, Pulses w/18 Parameters	**25**	momi2	15.29	55.88	–
		momi3 (CPU)	7.27	17.61	1.5e−06
		momi3 (GPU)	**0.32**	**0.56**	**1.4e−06**

a Fifteen CPUs were used in each CPU test and a single GPU was used in each GPU test. Runtime ∇ℓ is the total computation time for the derivative of all variables and the likelihood.

The bold values are the column minimum within each demographic model group.

First, we consider the performance improvement of momi3 relative to its predecessor momi2. For constant size models (demographies that begin with *xD*), momi3 running on a single GPU is more than 10 times faster in likelihood calculation, and up to 20 times faster in gradient calculation. For models featuring pulse migrations, the improvements are even more pronounced, e.g. a 30-fold improvement OOA-5D-Pulses demography. Less dramatic performance improvements on the order of 2–5× over momi2 were also observed even when we ran momi3 on CPU only, due to the efficiency gains of JIT compilation.

Next we turn to comparison with moments. In general, the simulation results show that large efficiency gains are again possible using momi3, however the improvements are not uniform. The largest differences were observed when comparing the time needed to evaluate the gradient of the 3D model with sample sizes n=200, where momi3 was about 1000-fold faster than moments. This is because diffusion-based methods compute all entries of the joint frequency spectrum, whereas momi and other coalescent-based approaches compute them entrywise, so they can exploit sparsity in the observed SFS. For instance, in the 3D model with n=200 samples per deme, moments computes all $201^{3}\approx$ 8 million SFS entries, even though only about $10^{4}$ distinct SFS entries are observed in a chromosome-length simulation.

**Figure 3:**
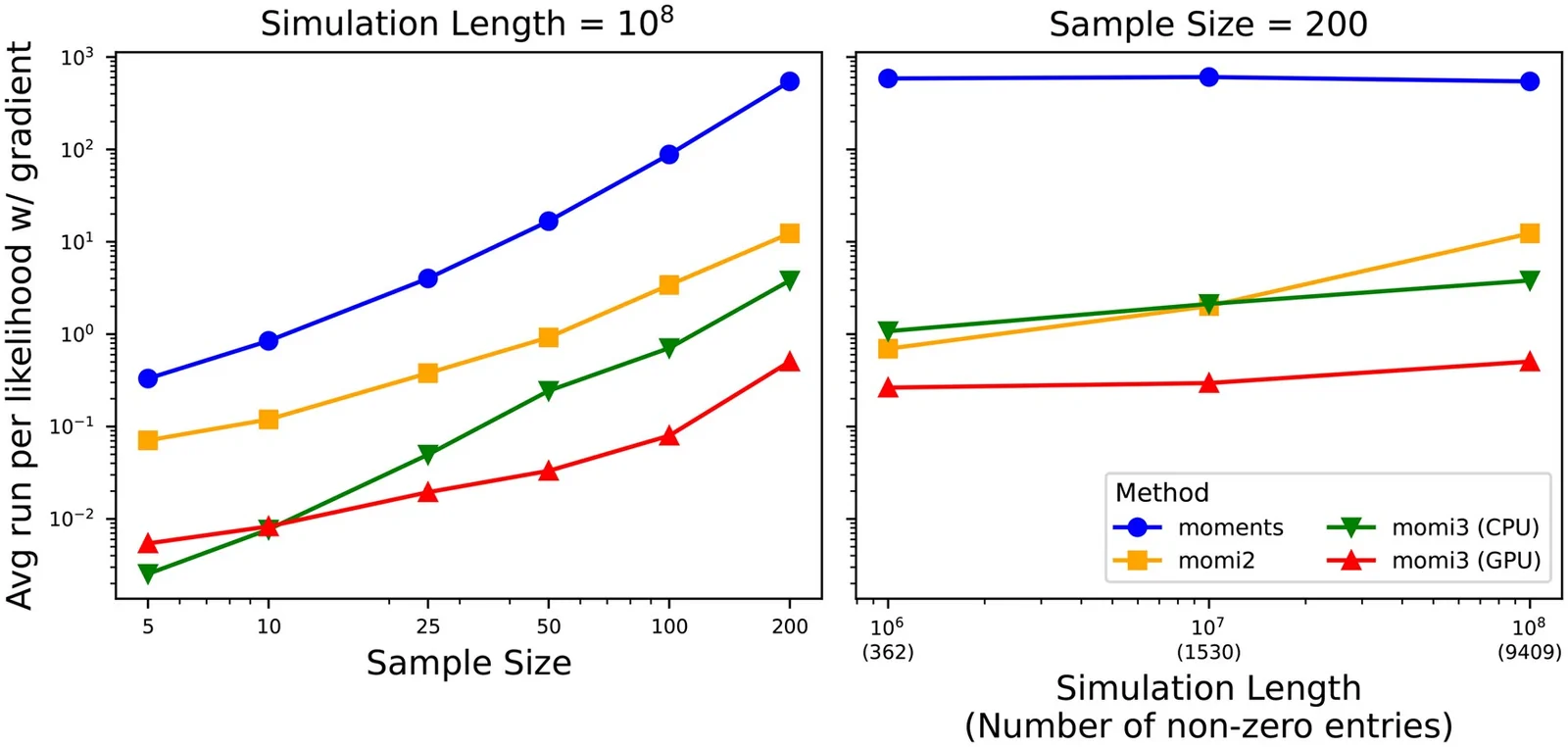
Runtime of a likelihood with gradient for the demography *3D w/5 Parameters* (see Fig. 1). The figure shows how each method scales by sample size (left) and simulation length (right).

**Figure 4:**
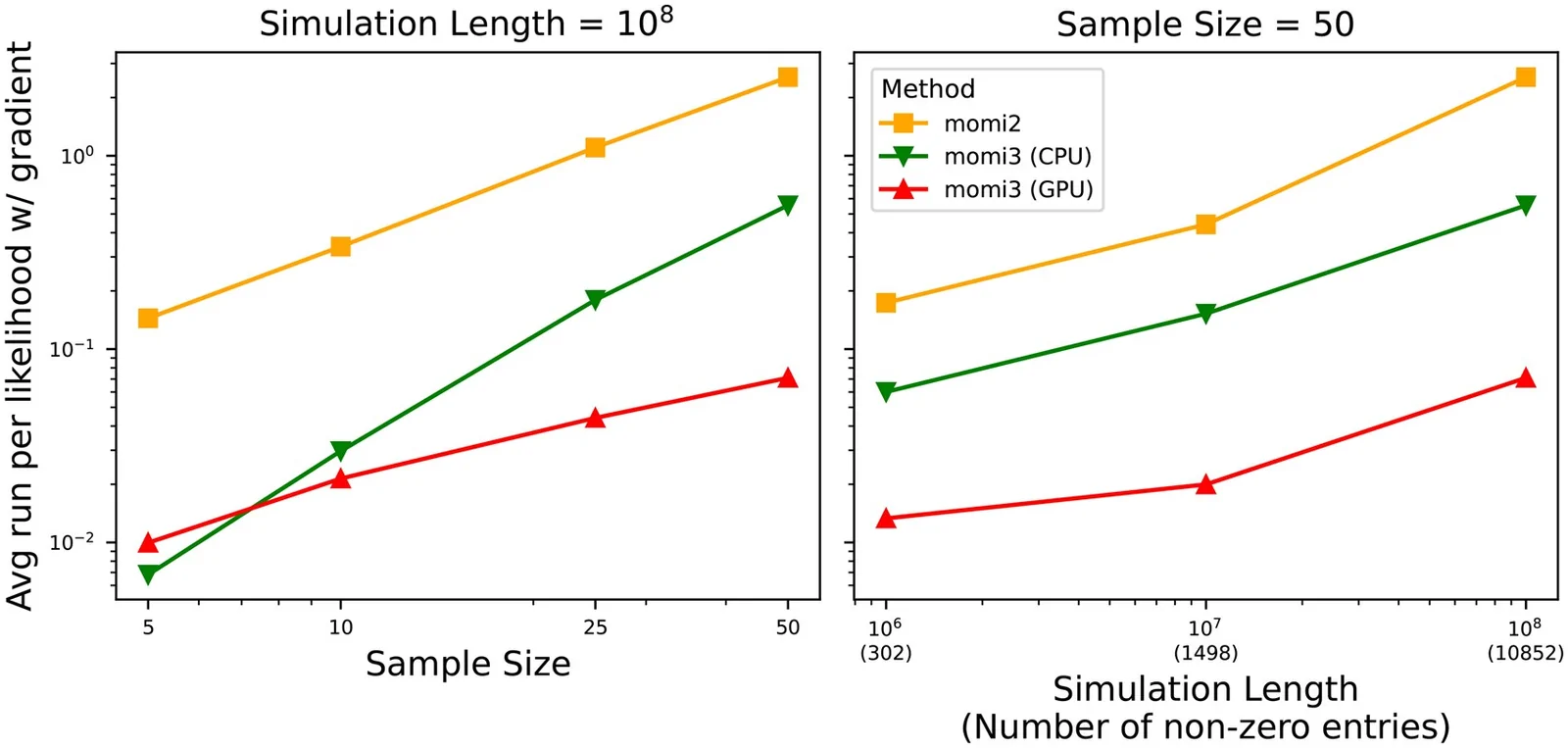
Runtime of a likelihood with gradient for the demography *4D w/6 Parameters* (see Fig. 1). The figure shows how each method scales by sample size (left) and simulation length (right).

**Figure 5:**
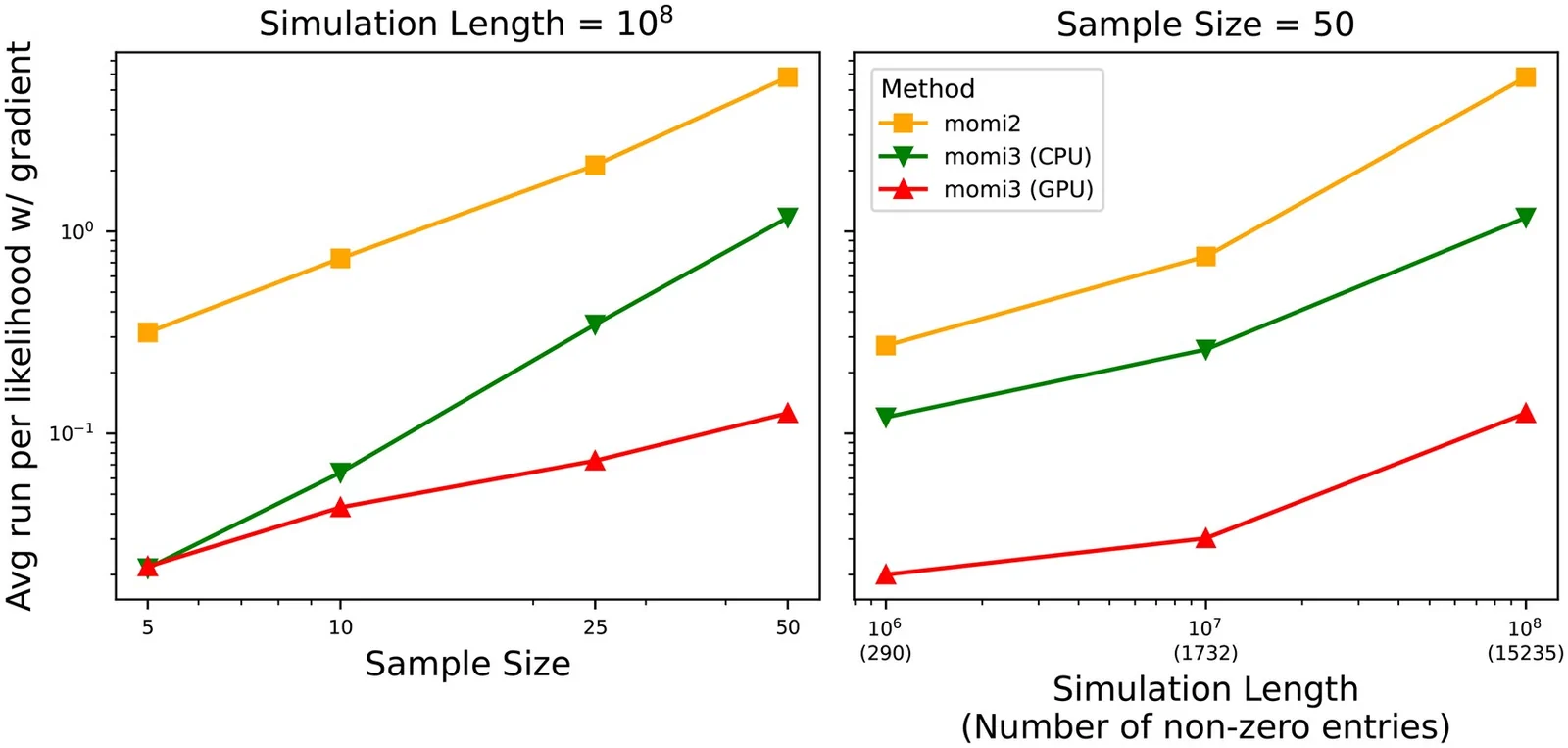
Runtime of a likelihood with gradient for the demography *5D w/7 Parameters* (see Fig. 1). The figure shows how each method scales by sample size (left) and sequence length (right).

Figure 3 shows how runtime increases for each method on this demography as both sample size and the number of SFS entries increase. In Figs 4 and 5, we repeated this analysis for larger 4*D* and 5*D* models, but we were not able to include moments, since with e.g. five demes it would need to compute $\approx 3.5\times 10^{8}$ SFS entries. Overall, for simple demographies that do not have admixture or migration, momi3 running on GPU can be expected to perform exceptionally well compared to moments.

**Figure 6:**
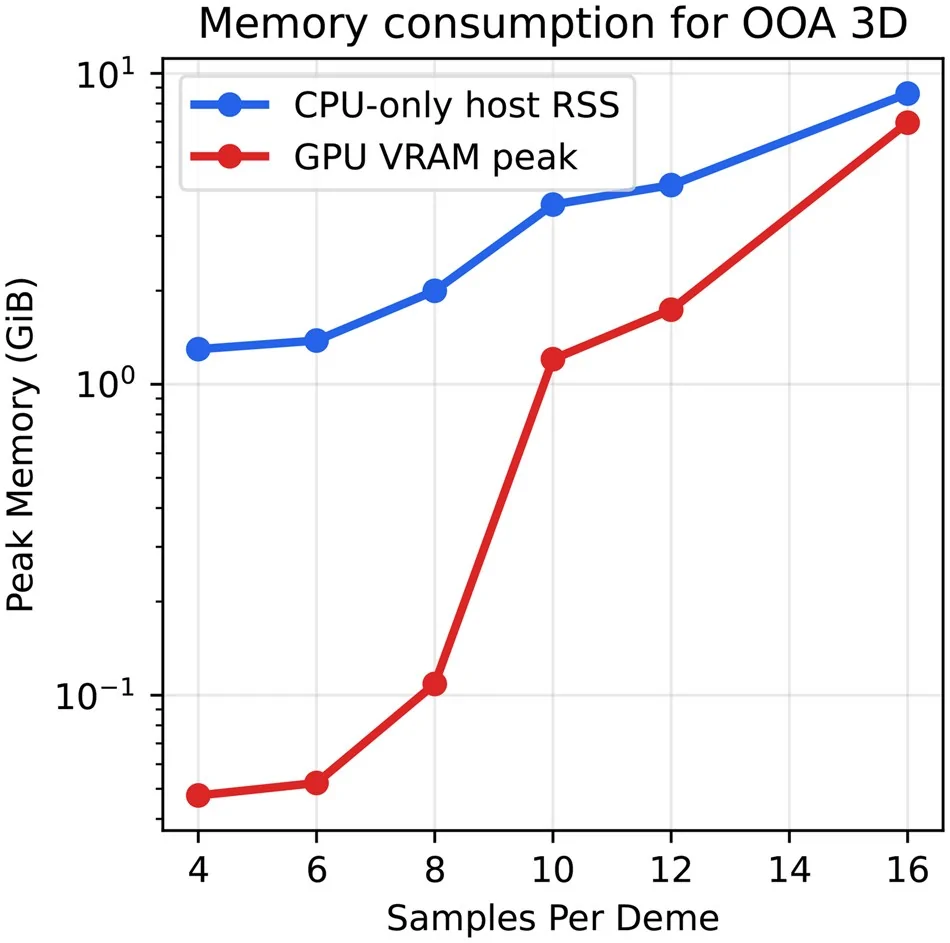
Memory consumption of a likelihood with gradient for the demography *3D w/5 Parameters* (see Fig. 1). The figure shows memory consumed by CPU and GPU modes.

We also studied memory usage across both CPU and GPU modes (Fig. 6). On a sample demography, peak memory usage scaled approximately polynomially with sample size, which is expected since the computations performed in momi3 involve a tensor of roughly size $O(n^{d})$. CPU memory consumption appears to be a constant factor higher than GPU, however CPU mode typically has access to much larger amounts of system memory than current GPU accelerators.

For models involving admixture or migration, we found less dramatic improvements, but in most cases momi3 was the fastest method. The biggest differences were observed when computing the likelihood+gradient, where for complex models (e.g. OOA 5D with either pulse or continuous migrations), momi3 was hundreds of times faster than moments; this was mainly attributable to the inherent speed advantage of automatic (momi3) over numerical (moments) differentiation. However, there were also examples such as OOA 3D where moments was faster than momi3 even though the latter was running on a GPU. For continuous migration models, most of the execution time for both methods is spent solving differential equations via sparse matrix multiplication. Support for sparse matrix routines in JAX is currently experimental, and we expect that the performance of momi3 will continue to improve as JAX matures.

#### 3.1.2 Numerical accuracy

The last column of Table 3 shows the numerical accuracy of each of the methods. For the accuracy metric, we used total variation (TV) distance between the computed and true expected frequency spectrum (viewed as a probability distribution over allele configurations). Since TV is between 0 and 1, this can be interpreted as percent difference between the two distributions. To compute the true frequency spectrum, we must of course rely on another computational method, chosen to minimize numerical error. For demographies that do not involve continuous migration, we used the values returned by momi2 as the reference, since the method is exact for that class of models. For demographies involving continuous migration, we followed moments. For speed and memory considerations, all computations were performed using 32-bit floating point numbers. Users who desire greater precision can enable double precision (64-bit mode) using an environment variable.

The results indicate that momi3 typically had the lowest numerical error for demographies involving pulse migrations. In fact, the difference between momi3 and momi2 was usually about $O(10^{-8})$, which is the limit of floating point accuracy when using 32-bit floating point numbers, the default mode for momi3. For larger, more complex demographies the error was sometimes as high as $O(10^{-6})$, which is still minuscule and should not affect most inference problems. These results are expected because momi2 and momi3 are algorithmically equivalent for this class of demographies, even though their implementations differ.

For demographies that have continuous migration, momi3 had error of about 1%−3%, depending on the complexity of the demography. This was about an order of magnitude larger than the difference between moments and ∂a∂i. We expected greater numerical differences to emerge in this setting because the method of calculation is fundamentally different; momi3 needs to compute a derivative (see Supplementary Proposition 1, available as supplementary data at *Bioinformatics* online), which will tend to introduce numerical inaccuracy compared to methods that do not. Potential users of momi3 should keep this in mind, and decide whether somewhat lower accuracy is compensated for greater speed and scalability.

#### 3.1.3 Compilation time

Finally, we benchmarked the compilation time of momi3 for each of the demographies listed above. As noted above, JIT compilation is unique to momi3 and a source of large efficiency gains. However, the compilation can require several minutes depending on the complexity of the demography. Enabling gradient mode also further increases compilation time. Table 4 shows compilation time (in seconds) for each demography, combination of likelihood or likelihood+gradient, and backend (CPU or GPU). For models that do not involve continuous migration, compilation times are quite reasonable; even the largest models we analyzed, containing hundreds of samples and many demes, compiled in under a minute. Compilation times tended to be slightly higher for GPU than CPU, and significantly higher for models that feature continuous migration. In the latter case, a lot of additional code is added including an ordinary differential equation (ODE) solver and many instances of (experimental, unoptimized) sparse matrix multiplication. Efforts to reduce compilation times for JAX models (e.g. by caching compilation results and modularizing subroutines) are currently underway, so we expect that compilation times will improve in the future.

**Table 4:** Compilation time of momi3.

Demographic model	*n*	ℓ **–CPU**	ℓ **–GPU**	∇ℓ **–CPU**	∇ℓ **–GPU**
2D w/4 Parameters	5	0.32	0.56	3.24	4.51
	10	0.37	0.57	3.69	4.31
	25	0.36	0.65	3.57	4.56
	50	0.44	0.70	4.06	4.84
	100	0.55	0.70	4.05	4.82
	200	0.89	0.78	4.71	5.87
3D w/5 Parameters	5	0.44	0.76	3.79	5.16
	10	0.50	0.79	3.97	5.00
	25	0.53	0.91	4.11	5.31
	50	0.78	0.92	4.73	5.80
	100	1.00	0.99	5.07	6.87
	200	2.58	1.41	8.50	13.13
4D w/6 Parameters	5	0.59	0.96	4.26	5.63
	10	0.68	1.01	4.75	5.72
	25	0.77	1.18	4.70	6.59
	50	1.20	1.16	5.78	7.05
5D w/7 Parameters	5	0.76	1.20	4.87	6.40
	10	0.85	1.22	5.08	6.42
	25	1.03	1.41	5.57	7.34
	50	1.72	1.47	7.07	9.01
OOA 2D, Migrations w/10 Parameters	5	–	9.83	–	62.67
	10	–	10.24	–	66.51
	15	–	10.57	–	66.31
	20	–	11.82	–	72.70
OOA 3D, Migrations w/13 Parameters	5	–	16.47	–	97.17
	10	–	18.45	–	107.65
	15	–	24.08	–	127.60
	20	–	43.08	–	232.78
OOA 4D, Migrations w/17 Parameters	5	–	40.09	–	222.25
OOA 5D, Migrations w/21 Parameters	5	–	78.36	–	396.78
OOA 5D, Pulses w/18 Parameters	3	4.57	6.02	15.84	20.93
	5	4.86	6.15	16.62	21.16
	10	5.49	6.23	18.47	22.87
	15	6.51	8.51	21.03	28.49
	25	12.56	8.27	37.73	40.71

### 3.2 Real data applications

Next, we turn to some applications of momi3 on real data. As shown in the preceding section, momi3 basically produces the same output as existing methods, but is faster and able to analyze larger samples. The papers describing those methods already performed extensive real data analysis, and we do not expect to find anything new by reproducing their results. We therefore focused primarily on analyzing real data problems that are out of the reach of existing methods. In what follows, we assumed a mutation rate of $1.29\times 10^{-8}$/bp/generation, allowing us to estimate event timings on an absolute scale, and utilized genomewide data from each human sample.

#### 3.2.1 Performance of the approximate method

**Figure 7:**
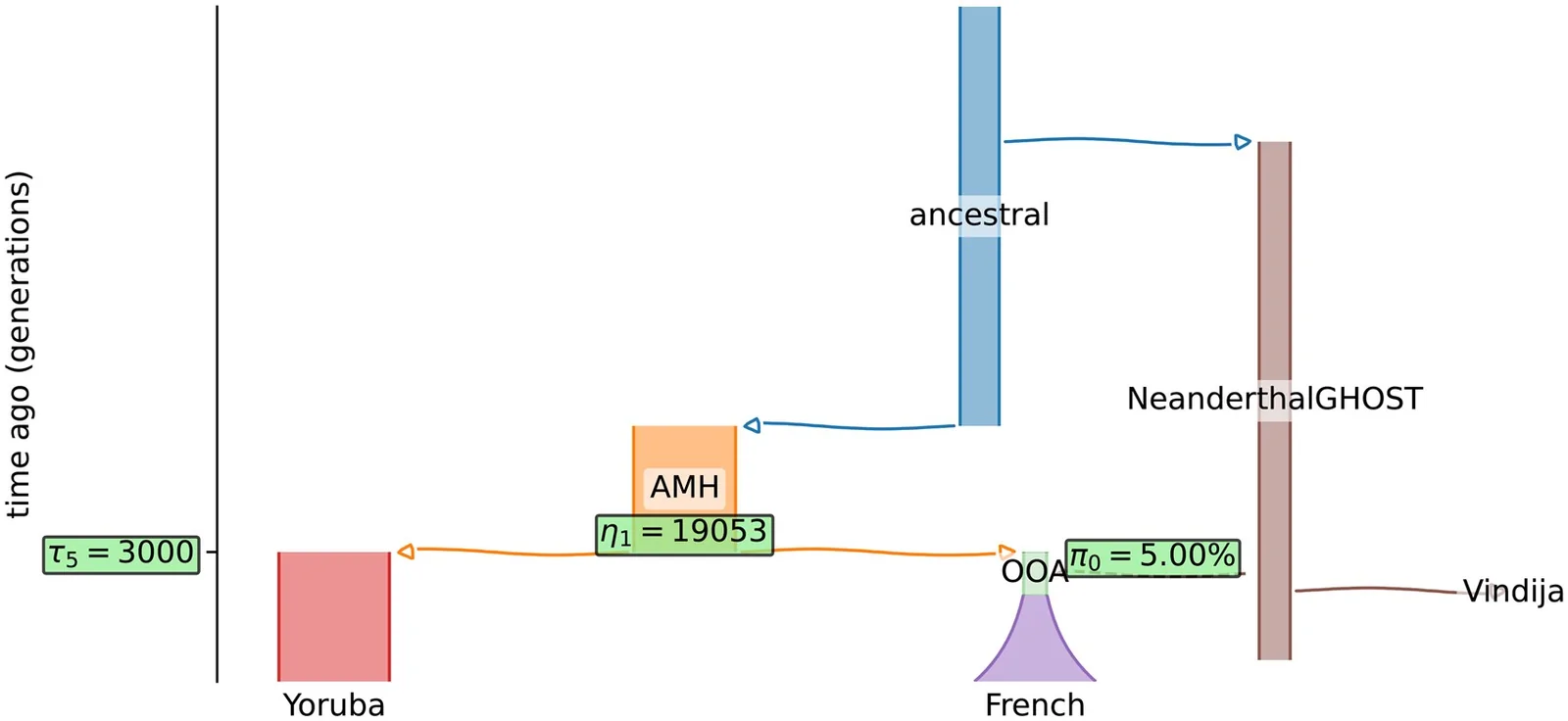
Out-of-Africa demography with Neanderthal admixture.The boxed variables are trained, while the remaining parameters were fixed. The trained variables were: $\tau_{5}$, the time of the Out-of-Africa event; $\eta_{1}$, the effective population size of Ancient Modern Humans; and $\pi_{0}$, the admixture fraction of Neanderthal in the OOA population.

First, we show how the approximation strategy outlined in Section 2.2.2 can be used to greatly reduce the computational burden of analyzing complex demographies using many samples. For this section we analyzed an out-of-Africa model with Neanderthal admixture (Fig. 7). We fit this model using all of the available samples in the dataset published by Wohns *et al.* (2022): 107 Yoruba, 28 French, and 1 Vindija Neanderthal genomes, for a total (haploid) sample size of 272. For simplicity, we used data from chromosome 20, and focused on inferring three parameters: $\tau_{5}$, the time of the out-of-Africa event; $\eta_{1}$, the effective population size of Ancient Modern Humans; and $\pi_{0}$, the admixture proportion of Neanderthals into the out-of-Africa (OOA) population.

Due to the large number of pulse admixtures, computations involving the exact model are slow. The first row of Table 5 (“No Sampler”) shows that, after a 26-s compilation step, each evaluation of the likelihood and its gradient takes roughly 3.1 s. Even for this simple, 3-parameter model, maximizing the likelihood requires several hundred gradient steps, so fitting time is several minutes overall. To quantify uncertainty by bootstrapping, we would need to repeat this step hundreds of times, requiring a large amount of computational resources.

**Table 5:** Runtimes using genealogical pruning.

Percentile	Compilation time	**Runtime** ∇ℓ
No Sampler	26.555	3.145
1.0	6.727	0.068
0.999	6.520	0.052
0.99	6.290	0.045
0.95	6.602	0.043

**Figure 8:**
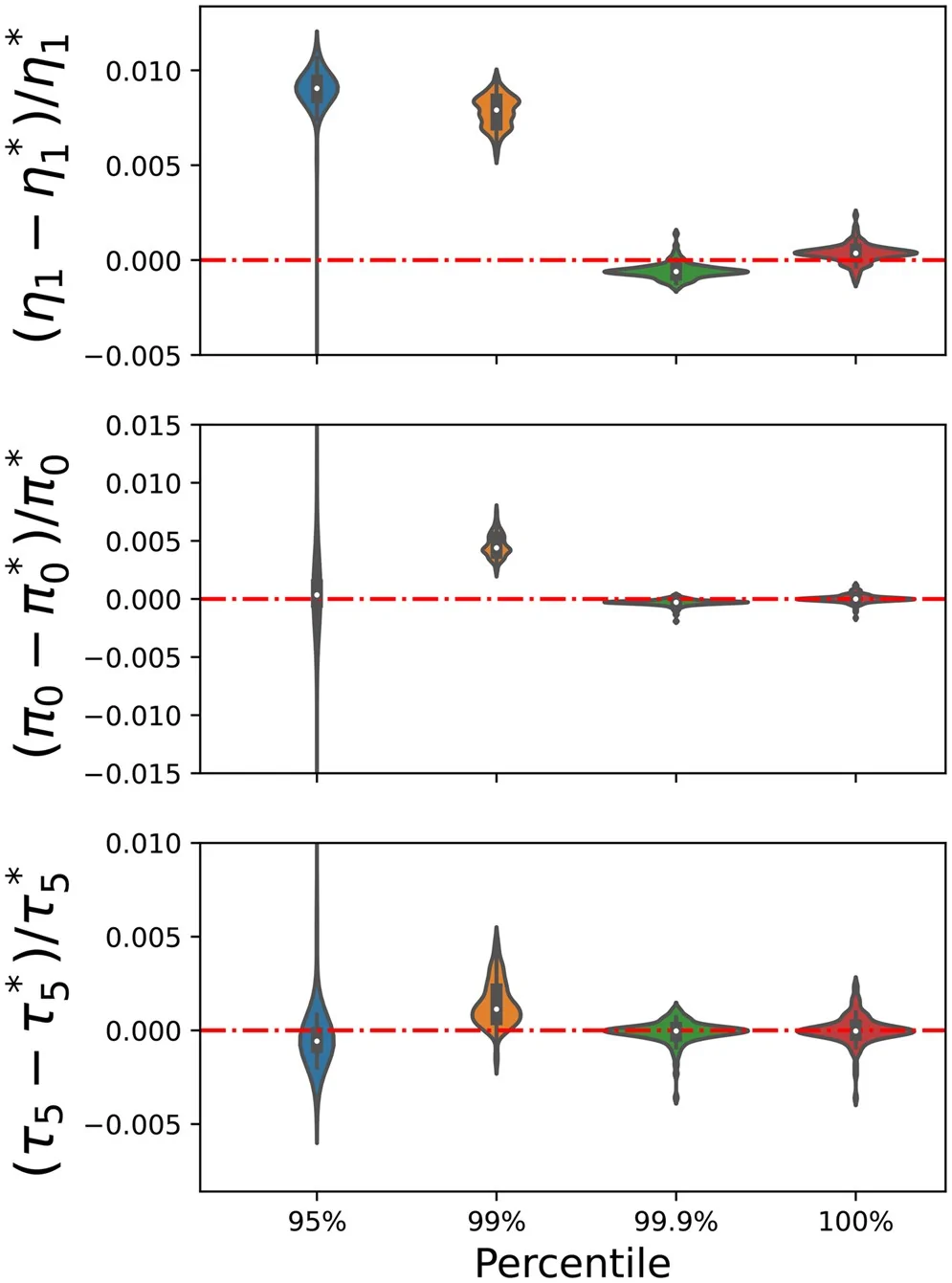
Distribution of the bias of estimates from the Fig. 7 model. Distributions are obtained from bootstrap samples. Star denotes the no-sampler estimate for the given bootstrap sampler. Closer to the broken line is better.

**Figure 9:**
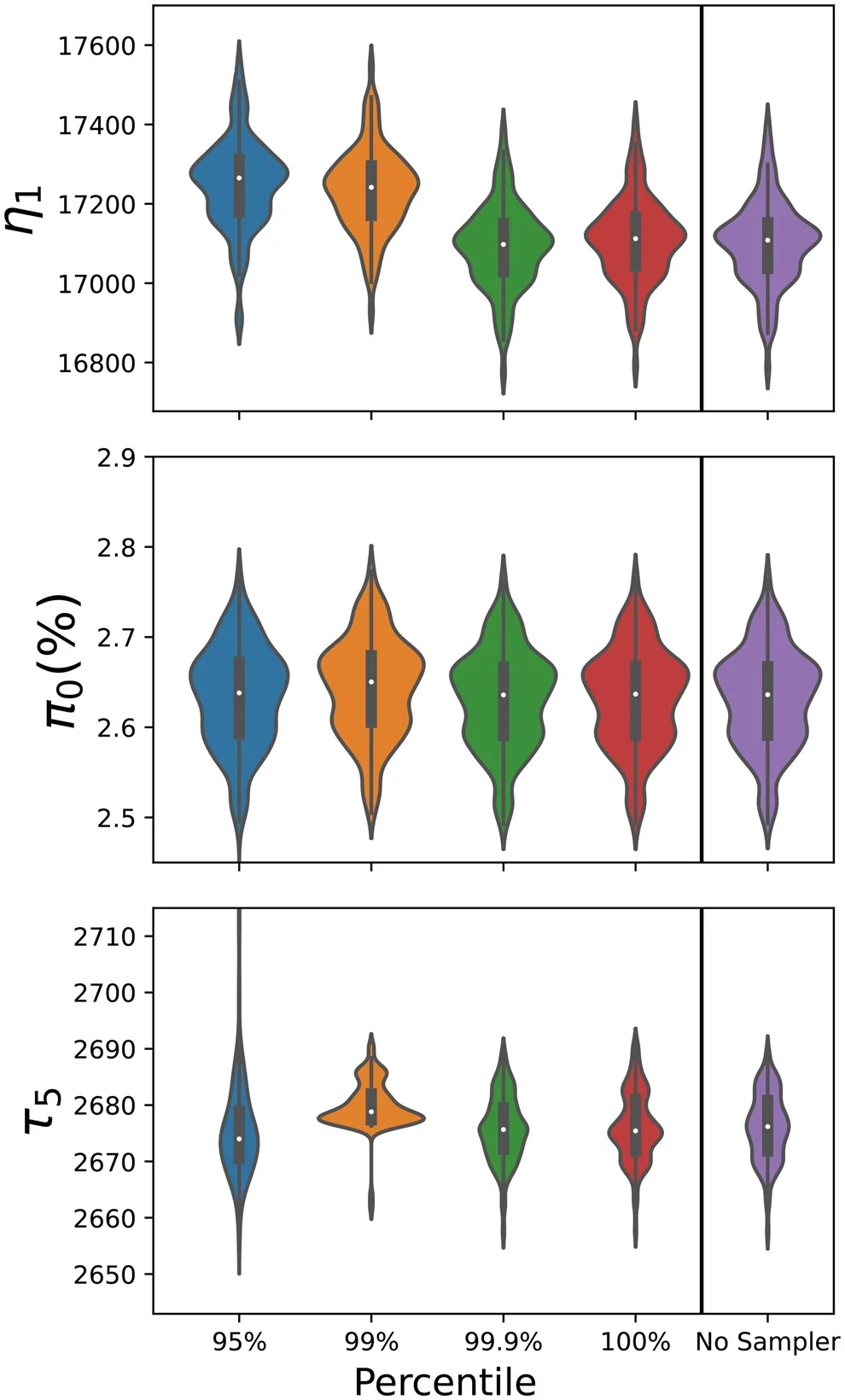
Distribution of the estimates from the Fig. 7 model. Distributions are obtained from bootstrap samples. Each percentile is used by genealogical pruning and compared with the no-sampler estimate.

This motivated us to study how Supplementary Proposition 2, available as supplementary data at *Bioinformatics* online could be applied to improve performance. For each tolerance level ϵ∈{0,0.001,0.01,0.05}, we performed Monte Carlo simulations to estimate the 1−ϵ quantile for the number of surviving ancestral lineages at each node in the event tree representation of this demography, and then used the built-in downsampling option of momi3 to perform approximate inference. This strategy produced a speedup of roughly 50–75× over the exact method (Table 5 rows 2–5), with larger ϵ leading to greater speedups. (Note that, due to Monte Carlo error, setting ϵ=0 (i.e. the max over all Monte Carlo trials) still results in performance improvements, and is not equivalent to the “No Sampling” case.) We then assessed the bias and variance of the estimated ${\hat{\tau }}_{5}$, ${\hat{\eta }}_{1}$, and ${\hat{\pi }}_{0}$. Here bias is defined as the difference between the maximum likelihood estimate using the exact model, versus each of the downsampled models, across a large number of bootstrap resamples from the observed frequency spectrum. Figure 8 shows the distribution of relative bias for each setting of ϵ (violin plots of each individual estimator are shown in Fig. 9). With 1−ϵ≥0.999, estimates using the approximate models are virtually indistinguishable from the exact model across all parameters. However, larger values of ϵ introduced more error. For $\eta_{1}$, using 1−ϵ≤0.99 produced a bias of about 1%, equivalent to about 150 units of $N_{e}$, and similar results were observed for estimating the admixture fraction $\pi_{0}$. Interestingly, the divergence time estimate $\tau_{5}$ seemed to be relatively less affected by downsampling, with mean bias of only about 0.1%.

In summary, using large values of ϵ (particularly ϵ=0.05) introduces additional bias and variability, and is probably best avoided except as a last resort. However, setting ϵ=0.001 (or even ϵ=0) resulted in a substantial efficiency gain, with minimal impact on the parameter estimates.

#### 3.2.2 Inference of archaic admixture using many genomes

Encouraged by these results, we turned to a more complex demographic model of archaic admixture. As is well-known, non-African individuals carry about 1%–4% Neanderthal DNA due to past interbreeding between humans and Neanderthals. There is also evidence of admixture from Denisovans into modern humans. However, which model choices best capture the underlying population structure remains uncertain (Sankararaman *et al.* 2014, Vernot and Akey 2015, Dannemann and Racimo 2018). In particular, it is not clear if these admixture events are better modeled as a sequence of discrete pulses, or perhaps as low-level continuous gene flow over a period of time (Browning *et al.* 2018, Villanea and Schraiber 2019, Durvasula and Sankararaman 2020).

**Figure 10:**
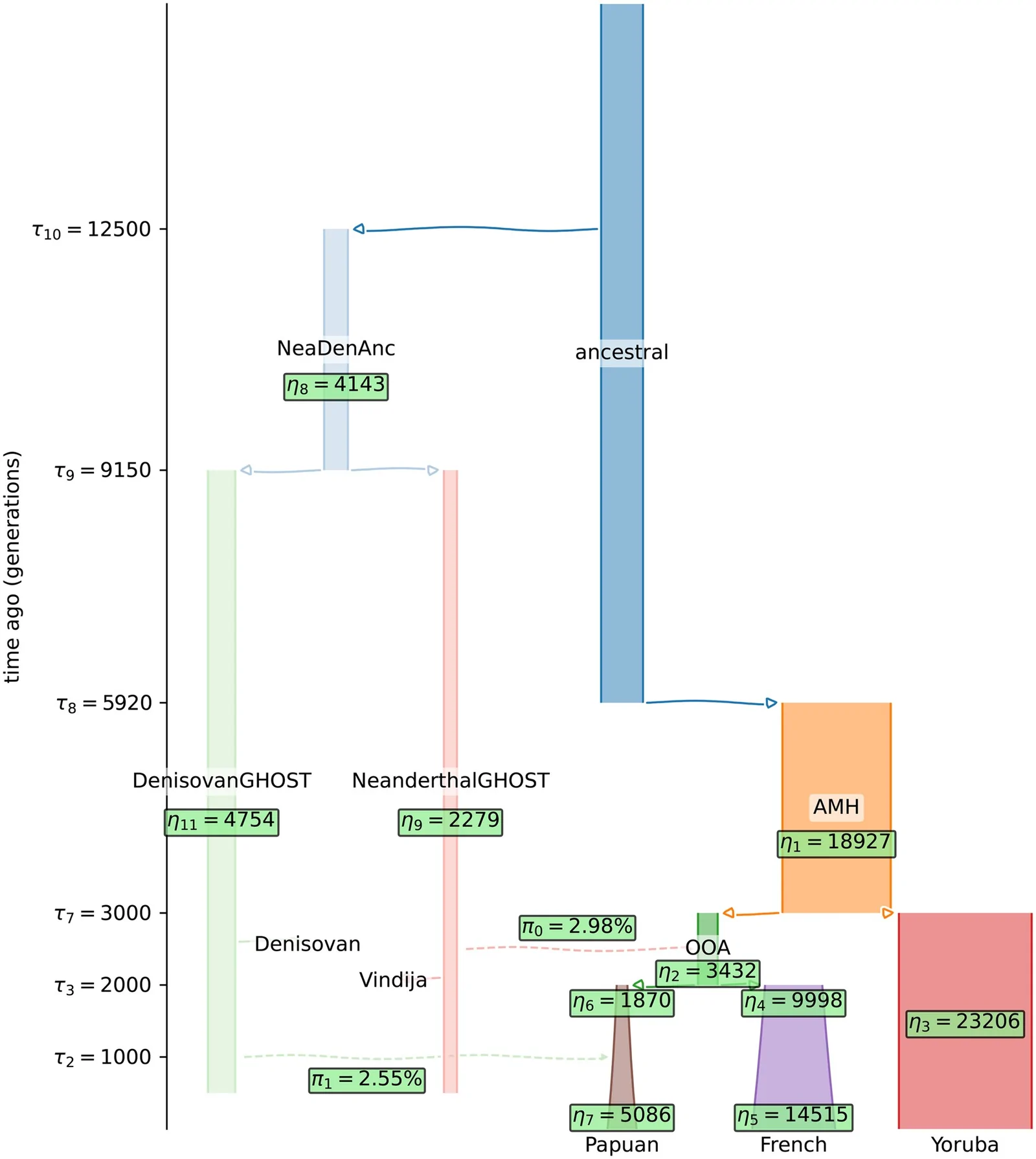
Out-of-Africa model with two extinct hominids. Parameter estimates are shown in boxes.

To study this question we used the demographic model proposed by Reich *et al.* (2010) and fit it to all the available data in Wohns *et al.* (2022) dataset (all sample sizes are diploid): 107 Yoruban samples, 28 French, 15 Papuan, as well a Vindija Neanderthal, and a Denisovan individual. Figure 10 shows a visualization of this demography.

We could not use either ∂a∂i or moments to analyze this model, since it contains more than five populations. Nor could we employ momi3 directly; attempting to do so exhausted all the available memory on our GPU. Thus, we turned to pruning, as in the preceding section, in order to make progress.

There are a total of 12 parameters in the model, summarized in Table 6. (We did not attempt to estimate time parameters, and fixed them to the values originally inferred by Reich et al.) One hundred non-parametric bootstrap replicates, formed by sampling with replacement from the observed frequency spectrum, were also performed in order to obtain confidence intervals. Each run took about ten minutes using a Tesla V100 GPU.

**Table 6:** Estimated parameters along with their nonparametric bootstrap confidence intervals for the demography in Fig. 10.

Parameter	Description	Estimate	2.5%	97.5%
$\eta_{1}$	Size of ancient modern humans	18 928	18 732	19 155
$\eta_{2}$	Size of out-of-Africa humans	3433	3371	3477
$\eta_{3}$	Size of Yoruba	23 207	23 040	23 363
$\eta_{4}$	Size of French after out-of-Africa	9998	9263	10 707
$\eta_{5}$	Recent size of French	14 516	13 744	15 450
$\eta_{6}$	Size of Papuan after out-of-Africa	1870	1788	1967
$\eta_{7}$	Recent size of Papuan	5087	4745	5350
$\eta_{8}$	Size of Neanderthal-Denisovan Ancestor	4143	4007	4262
$\eta_{9}$	Size of Neanderthal	2280	2201	2372
$\eta_{11}$	Size of Denisovan	4754	4370	5131
$\pi_{0}$	Neanderthal admixture proportion of OOA	2.9805%	2.8806%	3.0579%
$\pi_{1}$	Denisovan admixture proportion of Papuan	2.5456%	2.4277%	2.6663%

Results are shown in the right columns of Table 6, with point estimates superimposed on the demography visualization (Fig. 10) for convenience. In Reich *et al.* (2010), Vindija Neanderthal admixture is estimated between 1.3% and 3.7% and Denisovan admixture between 3.8% and 5.8%. Our confidence interval for the Neanderthal individual agrees with their *S*-statistic based confidence interval ($\pi_{0}$ at Table 6), however we find a lower admixture proportion from Denisovan to Papuans ($\pi_{1}$ at Table 6). Our findings agree with another study by Vernot *et al.* (2016), who estimated the proportion of Denisovan to Papuan admixture to be around 3% using $f_{4}$ analysis.

Next, we considered an extended model that also allowed for continuous gene flow between Neanderthals and anatomically modern humans. At present, the timing and nature of ancient human migration events, particularly those involving Neanderthals and modern humans, remain uncertain. To study this further, we added two additional parameters to the model considered above, which allows for a constant rate of asymmetric migration between AMH and Neanderthal between approximately 100 000 and 175 000 years ago.

**Figure 11:**
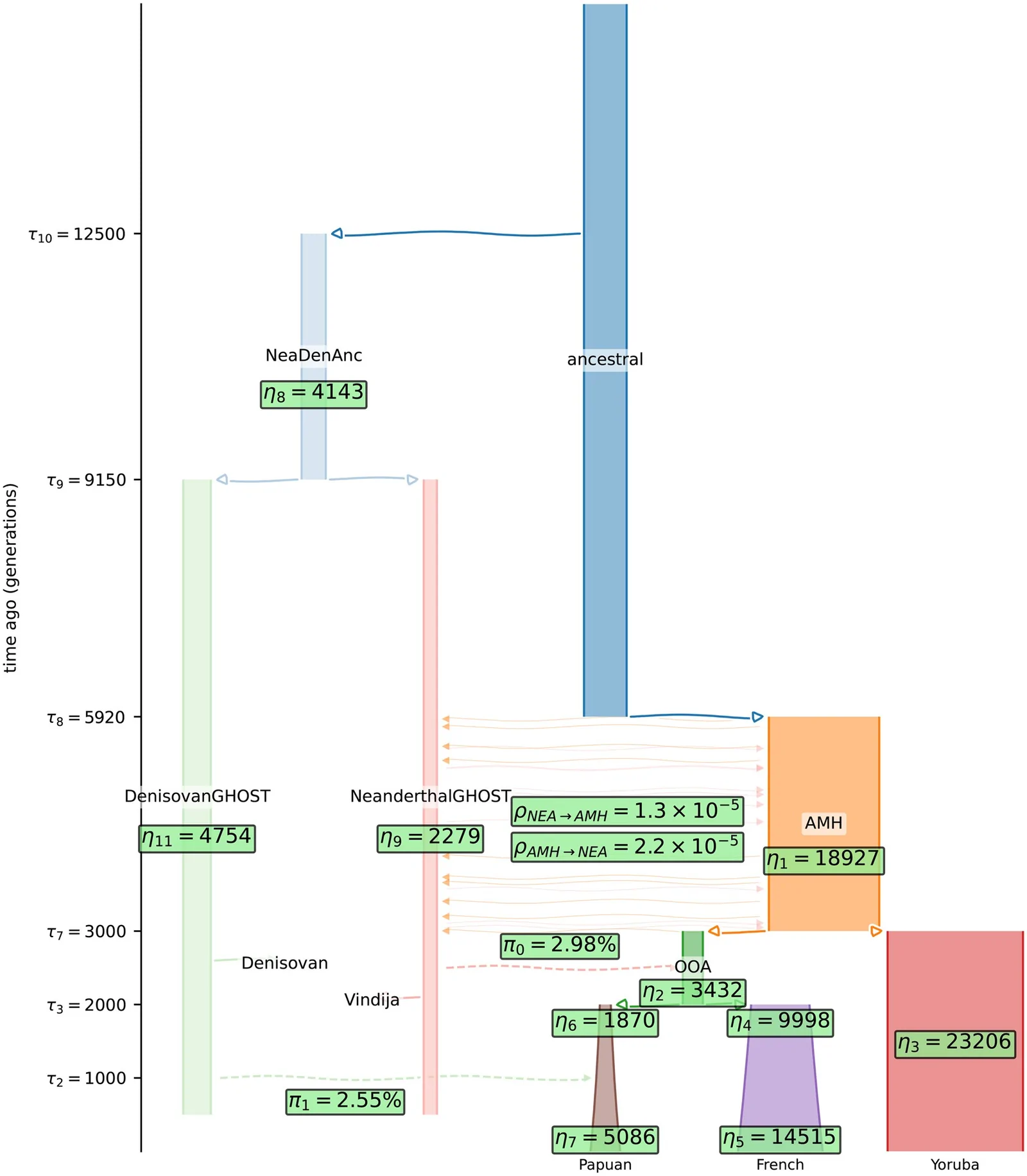
Out-of-Africa model with two extinct hominids and continuous gene flow between AMH and Neanderthals. Parameter estimates are shown in boxes. η parameters are effective population sizes (2 *N*), π parameters are admixture proportions, and ρ parameters are migration rates.

The extended model is shown in Fig. 11.

Adding continuous migration to the model requires considerably more computation. The time needed to evaluate the gradient of the log-likelihood jumped from around 1 s in the pulse-only model, to ∼200 s. This was too long for bootstrapping to be feasible, so instead we obtained maximum likelihood estimates by running a single optimization, and used asymptotic theory to form confidence intervals. Results are shown in Table 7.

**Table 7:** Estimated migration rates along with their parametric confidence intervals for the demography in Fig. 11.

Parameter	Description	Estimate	2.5%	97.5%
$\rho_{0}$	Migration rate from Neanderthal to AMH	1.25e−05	1.04e−05	1.46e−05
$\rho_{1}$	Migration rate from AMH to Neanderthal	2.24e−05	2.17e−05	2.31e−05

The migration rate from Ancient Modern Humans to Neanderthals ($\rho_{1}$) was found to be almost twice the rate of the other direction ($\rho_{0}$), indicating a greater gene flow from Ancient Modern Humans to Neanderthals. $\rho_{1}$ also had a narrower confidence interval compared to $\rho_{0}$.

**Figure 12:**
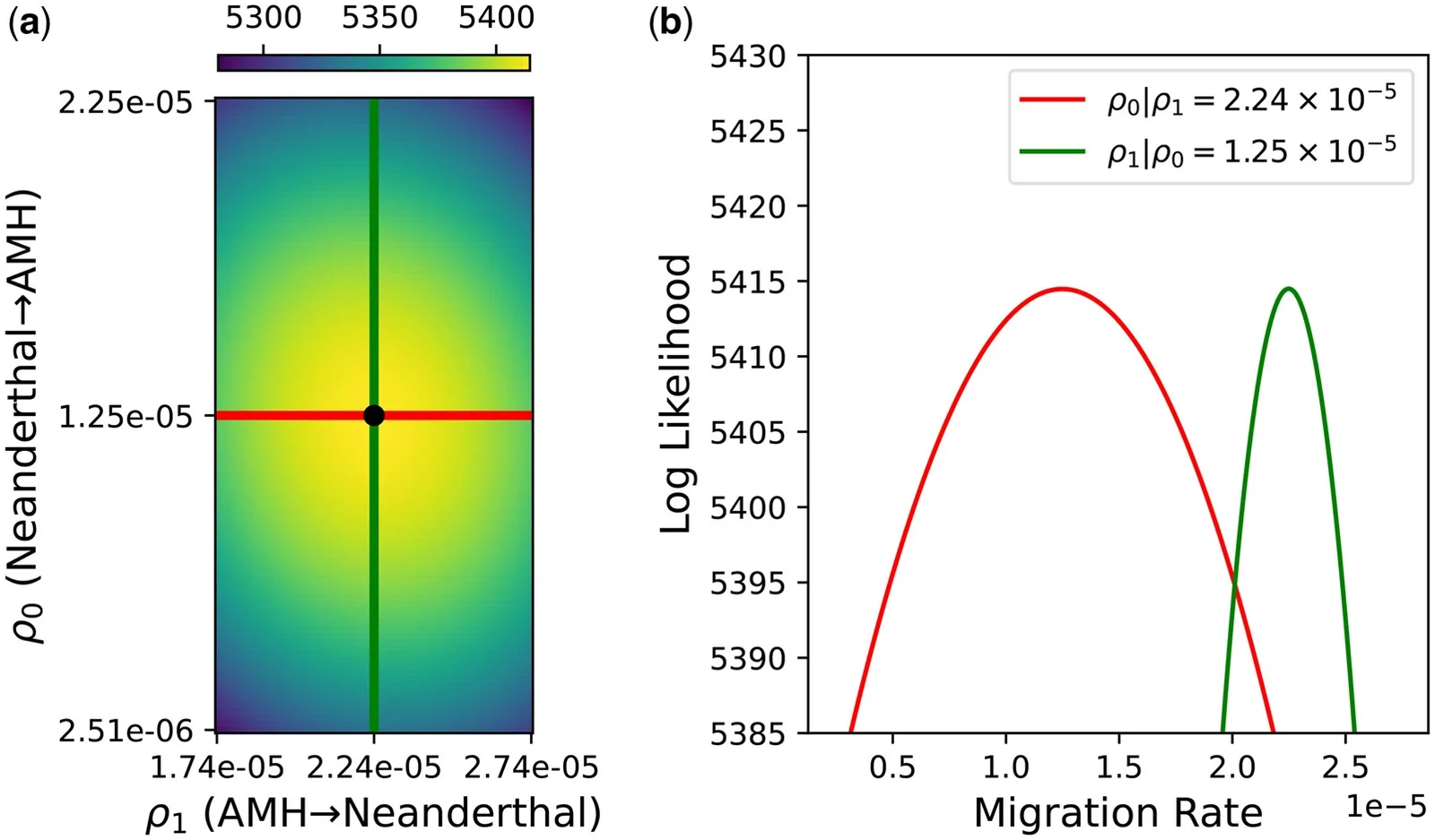
The likelihood surface of migration rates. Panel (a) evaluates the likelihood over a grid of migration rates and visualizes it as a heatmap, where lighter colors indicate higher likelihood. Panel (b) plots each migration rate against the likelihood while holding the other migration parameter fixed at its maximum likelihood estimate.

To better understand this asymmetry, we exploited momi3’s ability to quickly evaluate the log-likelihood function for many parameter values, and directly visualized the likelihood surface (Fig. 12a). The log-likelihood values were calculated on a grid of migration rates around their maximum likelihood estimates. The values of log-likelihoods represent the values subtracted from the log-likelihood without migration (value for $\rho_{0}^{*},\rho_{1}^{*}$ is equal to $\ell (\rho_{0}^{*},\rho_{1}^{*})-\ell (0,0)$). The green line represents the likelihood values while holding $\rho_{1}$ constant at $2.24\times 10^{-5}$, and the red line represents the likelihood values while holding $\rho_{0}$ constant at $1.25\times 10^{-5}$. We plotted these lines in Fig. 12b. We observed that $\rho_{1}$ had a sharper peak, resulting in a narrower confidence interval in Table 7. The flatter peak on $\rho_{0}$ (Neanderthal to AMH) indicates that even small values of $\rho_{0}$ are still relatively likely. Hence, the migration rate from Ancient Modern Humans to Neanderthals seems much more pronounced compared to the other direction.

## 4 Conclusion

We have described momi3, a method for inferring complex demographies from the JSFS of multiple populations. This work extends our earlier method momi2 in several ways. First, we added support for continuous migration, bringing the feature set of momi at least to parity with other methods in this space. Second, we improved on the existing state-of-the-art by implementing a novel pruning strategy, which prunes unlikely genealogies from the analysis, reducing memory and computational requirements for large sample sizes. Finally, we made various implementation and user interface improvements which make momi3 easier to use and able to run seamlessly on CPUs and GPUs.

On simulated data, momi3 is up to 1000× faster than existing methods, with more modest speed improvements depending on the nature of the underlying demography. We also applied momi3 to real data, analyzing multiple genomes to study archaic admixture in modern humans. We constructed a demography similar to the one studied by Reich *et al.* (2010) and fit it to all the available data in Wohns *et al.* (2022). To the best of our knowledge, no existing method could successfully fit such a model to a dataset of this size.

Despite these improvements, limitations remain. Under continuous migration between *d* demes, our method must integrate a *d*-dimensional system of ordinary differential equations, so realistically it can analyze around d=5 populations which continuously exchange migrants. Also, certain biological phenomena, namely linkage disequilibrium and natural selection, are not easily accommodated using the techniques we have developed here. Users needing to model these phenomena may need to resort to simulation-based approaches, which compute the expected frequency spectrum using a Monte Carlo approach (Excoffier *et al.* 2021).

There are several areas for future work. Due to the currently experimental support for sparse tensor operations in JAX, momi3 is not that much faster, if at all, than existing methods (in particular moments) for analyzing demographies that feature continuous migration between a large number of populations. Similarly, JIT compilation times using our method can be long: comparing Table 4 with Table 3, we see that compilation times are currently approximately 20 times slower than the actual runtimes. While the one-time cost of compilation is generally negligible in optimization problems that require running the optimizer multiple times until convergence, it becomes a significant issue when considering many topologically distinct demographies. At the time of this writing, efforts to reduce JIT compilation times in JAX are underway, and we believe that the issue may be ameliorated in future versions.

## Supplementary Material

btag511_Supplementary_Data

## Data Availability

All of the data analyzed in this paper are publicly available. momi3 is implemented as part of our library demestats, available at https://github.com/jthlab/demestats. Documentation and tutorials are available at https://demestats.readthedocs.org.
