# Supplementary material for: Faster inference of complex demographic models from large allele frequency spectra
Source: Bioinformatics. 2026 Jul 11;42(8):btag511. doi: 10.1093/bioinformatics/btag511 (PMC13472727; doi:10.1093/bioinformatics/btag511)
Supplement: Supplementary file 1 [file btag511_supplementary_data.pdf]

Supplementary Information: Faster inference of complex demographic models from large allele frequency spectra

Enes Dilber, Jiatong Liang, Junyan Tan, and Jonathan Terhorst

Supplemental Information

In this section we formally define and prove the correctness of the continuous migration and genealogical pruning described in the main text.

0.1 Summary of momi2

First, we need to review some salient technical details about how its predecessor works. MOMI2 decomposes an arbitrary demographic model containing splits, mergers, pulse admixture events, and population size changes, into a data structure called an *event tree*. Probabilistic message-passing (Wainwright and Jordan, 2008) is then performed on this tree in order to compute the likelihood of a given configuration of ancestral and derived alleles sampled at the tips of the demographic model. A complete technical description of the MOMI2 algorithm can be found in Kamm et al. (2020). We follow their notation (see Table 1 of that paper for a guide) in the sequel to streamline the exposition and minimize confusion.

To compute the expected frequency spectrum, MOMI2 requires two quantities. First, there is the partial likelihood tensor $\ell_{\mathbf{x}}^{E,\mathbf{t}}$, which, for a given node E in the event tree, gives probability of all of the data in the clade subtended by E, conditional on the state $\mathbf{x}$ of the ancestral populations at E. The second is the "truncated SFS", $f_v(k)$, for a given population v, which is defined by the relationship

$$\mathbb{E}[\#\text{ of mutations at } v \text{ with } X_v = k] = \theta f_v(k) + o(\theta). \tag{1}$$

In words, $f_v(k)$ is the expected number of mutations, under an infinite sites model, that have k ancestral copies in v at the earliest (closest to the present) time at which v exists in the model. MOMI2 works by solving a dynamic program, denoted $\mathrm{DP}(\ell^1, \dots, \ell^p)$ (c.f. Kamm et al., 2020, Algorithm 2), in terms of $\ell_{\mathbf{x}}^{E,\mathbf{t}}$ and $f_v(k)$ for each population v and event E in the event tree representation of the demography.

0.1.1 Continuous migration

The fundamental quantity in MOMENTS is expected number of segregating sites which are observed $\mathbf{i} \in (n_1+1)\times\cdots\times(n_p+1)$ times in a sample of size $\mathbf{n} = (n_1, \dots, n_p)$ (denoted $\Phi_{\mathbf{n}}(\mathbf{i})$ in their paper.) As shown by Jouganous et al., as this tensor evolves forwards in time, it satisfies the system of ordinary differential equations

$$\dot{\Phi}_{\mathbf{n}}(\mathbf{i}) = \left[\mathcal{U}_{\mathbf{i}} + \sum_{j=1}^{p} \frac{1}{4N_j}\tilde{\Delta}_{n_j,i_j} + \sum_{k\neq j} m_{jk}\hat{\mathcal{M}}_{jk}\right]\Phi_{\mathbf{n}}, \tag{2}$$

where the multilinear operators $\tilde{\Delta}_{n,i}$, $\hat{\mathcal{M}}_{jk}$, and $\mathcal{U}_i$ are given by equation (A2), equation (A4) (using the jacknife approximation described in Appendix D), and Appendix B, respectively, of Jouganous et al. (2017).

As already noted by Gravel and co-authors, the MOMENTS quantity $\Phi_{\mathbf{n}}$ is quite related to the partial likelihoods tracked by MOMI2; they are, in , "dual" to each other. To see this, observe that the "drift operator" $\tilde{\Delta}_{n,i}$ in (2) is precisely the *transpose* of the Moran rate matrix utilized by MOMI2 (compare equations (A2) of Jouganous et al. with the definition of the rate matrix $Q = (q_{ij})$ immediately preceding equation (19) of Kamm et al., 2017). This is so because time runs oppositely in the two algorithms: MOMI2 transitions partial likelihood tensors *backwards* in time by right-multiplication with $e^{\mathbf{Q}}$, where $\mathbf{Q} = Q_1 \oplus \cdots \oplus Q_p$ is the Kronecker sum of Moran rate matrices corresponding to each population $i = 1, \ldots, p$. Similarly, MOMENTS transitions the expectation tensor $\Phi_{\mathbf{n}}$ *forwards* in time by solving the system (2), which reduces (up to scaling factors) to $\dot{\Phi}_{\mathbf{n}} = \mathbf{Q}^{\intercal}\Phi_{\mathbf{n}}$ in the case of constant population size and no mutation or migration.

For a given event tree node E, let K_E be the set of populations that exist at E, as in Kamm et al. (2020). To lift the partial likelihood of a set $M \subset K_E$ of populations experiencing continuous migration τ generations into the past, we solve the *adjoint* of (2) subject to the initial condition $\Phi_{\mathbf{n}}^{(0)} \equiv \ell_{\mathbf{x}}^{E,\mathbf{t}_0}$, where $\mathbf{t}_0 = \mathbf{t} - \tau \sum_{v \in M} \mathbf{e}_v$, to arrive at $\Phi_{\mathbf{n}}^{(\mathbf{t})} = \ell_{\mathbf{x}}^{E,\mathbf{t}}$. In practice this simply amounts to taking the transpose of each of the operators shown above and solving the resulting ODE system. To solve for the adjoint when all populations have constant size, we use a matrix-free method for computing the action of the matrix exponential (Al-Mohy and Higham, 2011) on the initial tensor $\ell_{\mathbf{x}}^{E,\mathbf{t}_0}$, which allows us to exploit the sparsity and/or Kronecker product structure of the operators $\tilde{\Delta}_{n,i}$, $\hat{\mathcal{M}}_{jk}$ and $\mathcal{U}_i$ via fast matrix-vector products. If one or more populations experiences growth, then the N_j in terms (2) are no longer constant, so we use a differential equation solver as in Jouganous et al..

The other quantity we require is the truncated frequency spectrum, denoted $f_v(k)$ above in the case of a single population v. To generalize this to the setting of continuous migration, we must compute the truncated frequency spectrum for multiple populations exchanging migrants—a new quantity not considered in MOMI2. To find it, we simply observe that the expectation on the right hand side of equation (1) is exactly the same as the quantity $\Phi_{\mathbf{n}}$ tracked by MOMENTS. Hence, differentiating the solution of the original system of ODEs obtained by MOMENTS with respect to the mutation rate parameter yields the multipopulation analog of the truncated frequency spectrum. Note that, in contrast to the lifting operation, here we solve the primal system forwards in time. The appropriate initial condition is thus $\Phi_{\mathbf{n}}^{(0)}(\mathbf{i}) = \mathbf{1}_{\{\mathbf{i}=\mathbf{0}\}}$, which ensures that only branch length arising from *de novo* mutations in the populations in question are considered in the expectation. The following proposition formalizes these arguments.

Proposition 1. *Let $\Phi_n^{(t)}(i)$ be the solution to the system* (2) *under a symmetric finite-genome mutation model (i.e. $\mu = \nu = \theta$ in the operator $\mathcal{U}_i$), with the initial condition is $\Phi_{\mathbf{n}}^{(0)}(\mathbf{i}) = \mathbf{1}_{\{\mathbf{i}=\mathbf{0}\}}$. The truncated frequency spectrum up to time τ for these populations equals*

$$\left.\frac{\partial \Phi_{\mathbf{n}}^{(\tau)}(\mathbf{i})}{\partial \theta}\right|_{\theta=0} \in \mathbb{R}^{(n_1+1)\times\cdots\times(n_p+1)}.$$

To compute the derivative displayed above, we can use either automatic differentiation (see below), or solve a related system of ODE's using the forward sensitivity method (e.g., Bartlett, 2008).

0.2 Genealogical pruning

The correctness of the pruning procedure follows from the following proposition. All notation is carried over from the preceding section. Additionally, for a population $v \in \{1, \dots, \mathcal{D}\}$ in the demography, let $C_k^v(t)$ denote the event that the population immediately ancestral to v has no more that k lineages ancestral at time τ. Then we have the following result:

Proposition 2. *Let $\ell_{\mathbf{x}}^{E,\mathbf{t}} \in \mathbb{R}^{(n_1+1)\times\cdots\times(n_p+1)}$ be a partial likelihood tensor, where $p = |K_E|$, and let $v \in K_E$ be a population included in the partial likelihood tensor. Then conditional on the event $C_k^v(t_v)$ that v has no more than k surviving ancestral lineages at time t_v, the output of $\mathrm{DP}(\ell^1, \dots, \ell^p)$ is unchanged if we make the substitution*

$$\ell_{\mathbf{x}}^{E,\mathbf{t}} \to (I_{n_1+1} \otimes \cdots \otimes B^\dagger \otimes \cdots \otimes I_{n_p+1})\ell_{\mathbf{x}}^{E,\mathbf{t}} \in \mathbb{R}^{(n_1+1)\times\cdots\times(k+1)\times\cdots\times(n_p+1)}, \tag{3}$$

where $B = (b_{ij}) \in \mathbb{R}^{(n_v+1)\times(k+1)}$ is a matrix whose (i,j)-th entry is the probability of sampling without replacement $j \in \{0, \dots, k\}$ derived alleles in the population immediately ancestral to v out of a total of $i \in \{0, \dots, n_v\}$ derived alleles segregating in v, and $B^\dagger$ denotes the Moore-Penrose pseudoinverse.

Proof. The correctness of the modified algorithm follows directly from Lemmas 4 and 7 of Kamm et al. (2020): although those results are stated in terms of the partial likelihood of the leaf nodes ($\mathbf{X}_{\mathrm{Leaves}(E)}$ in their notation), by the Markov structure of the multipopulation coalescent, they also apply if we consider the partial likelihoods at internal nodes of the event tree. Hence, conditional on $C_k^v(t_v)$, the population immediately ancestral to v has at most k descendants, so by Lemma 7, its partial likelihood is independent of allele counts of the remaining lineages $\{k+1, \dots, n_v\}$. Finally, the combinatorial relationship between $\ell_{\mathbf{x}}^{E,t}$ the partial likelihood of the population immediately ancestral to v is given by Lemma 4 (see also the discussion immediately preceding the statement of the lemma). □

0.2.1 Correctness of the pruned lifting operation

A lifting operation (cf. Lemma 1 of Kamm et al., 2020) involves transitioning a partial likelihood from a more recent to more ancient time using the Moran genealogical process. Suppose we are lifting a particular population v, with effective size $N_v(t)$ and sample size n_v, from time t to time $t + \Delta t$. Define

$$\tau = \int_t^{t+\Delta t} \frac{ds}{N_v(s)}$$

to be the amount of scaled coalescent time that elapses during this operation. Finally, let $\mathcal{A}_n(t)$ denote the coalescent ancestral process associated to this population, i.e. a pure death process with $\mathcal{A}_n(0) = n$ almost certainly, and death rate $\mu_i = i(i-1)/2$ when the process is in state i. To find k and ϵ as above, we simulate[1] the coalescent ancestral process a large number of times, and find the $1-\epsilon$ quantile of the random variable $A_{n_v}(\tau)$. The correctness of this procedure follows directly from formula (2.1) of Griffiths and Tavaré (1998).

[1] Instead of simulating, we could also employ exact expressions for the distribution of $\mathcal{A}_n(t)$ (Tavaré, 1984), however these are known to be numerically unstable for large n.

References

A. H. Al-Mohy and N. J. Higham. Computing the action of the matrix exponential, with an application to exponential integrators. *SIAM Journal on Scientific Computing*, 33 (2):488–511, 2011.

R. Bartlett. A derivation of forward and adjoint sensitivities for odes and daes. Technical report, Tech. rep., Tech. Rep. SAND2007-6699, Sandia National Laboratories, 2008.

R. Griffiths and S. Tavaré. The age of a mutation in a general coalescent tree. *Communications in Statistics. Stochastic Models*, 14(1-2):273–295, 1998.

J. Jouganous, W. Long, A. P. Ragsdale, and S. Gravel. Inferring the joint demographic history of multiple populations: Beyond the diffusion approximation. *Genetics*, 206(3):1549–1567, July 2017. doi: 10.1534/genetics.117.200493.

J. A. Kamm, J. Terhorst, and Y. S. Song. Efficient computation of the joint sample frequency spectra for multiple populations. *J. Comput. Graph. Stat.*, 26(1):182–194, Feb. 2017. doi: 10.1080/10618600.2016.1159212.

J. A. Kamm, J. Terhorst, R. Durbin, and Y. S. Song. Efficiently inferring the demographic history of many populations with allele count data. *J. Am. Stat. Assoc.*, 115(531):1472–1487, 2020. doi: 10.1080/01621459.2019.1635482.

S. Tavaré. Line-of-descent and genealogical processes, and their applications in population genetics models. *Theoretical Population Biology*, 26(2):119–164, 1984. doi: 10.1016/0040-5809(84)90027-3.

M. J. Wainwright and M. I. Jordan. *Graphical models, exponential families, and variational inference*. Now Publishers Inc, 2008.
